# Acetylsalicylic Acid Resistance in Patients with Type 2 Diabetes Mellitus, Prediabetes & Non-Diabetic Coronary Artery Disease

**DOI:** 10.12669/pjms.303.4773

**Published:** 2014

**Authors:** Mustafa Cetin, Emrullah Kiziltunc, Zehra Guven Cetin, Hulya Cicekcioglu, Muslum Sahin, Serhat Isik, Alparslan Kurtul, Ender Ornek, Feridun Vasfi Ulusoy

**Affiliations:** 1 Mustafa Cetin, MD, Department of Cardiology, Ankara Numune Education and Research Hospital, Talatpasa Bulvari, 06100, Sihhiye, Ankara, Turkey.; 2 Emrullah Kiziltunc, MD, Department of Cardiology, Ankara Numune Education and Research Hospital, Talatpasa Bulvari, 06100, Sihhiye, Ankara, Turkey.; 3 Zehra Guven Cetin, MD, Department of Cardiology, Ankara Numune Education and Research Hospital, Talatpasa Bulvari, 06100, Sihhiye, Ankara, Turkey.; 4 Hulya Cicekcioglu, MD, Department of Cardiology, Ankara Numune Education and Research Hospital, Talatpasa Bulvari, 06100, Sihhiye, Ankara, Turkey.; 5 Muslum Sahin MD, Department of Cardiology, Ankara Numune Education and Research Hospital, Talatpasa Bulvari, 06100, Sihhiye, Ankara, Turkey.; 6 Serhat Isik, MD, Department of Endocrinology and Metabolism, Ankara Numune Education and Research Hospital, Talatpasa Bulvari, 06100, Sihhiye, Ankara, Turkey.; 7 Alparslan Kurtul, MD, Department of Cardiology, Ankara Education and Research Hospital, Sukriye Mahallesi, Ulucanlar Caddesi, 06340, Ankara, Turkey.; 8 Ender Ornek, Associate Professor, Department of Cardiology, Ankara Numune Education and Research Hospital, Talatpasa Bulvari, 06100, Sihhiye, Ankara, Turkey.; 9 Feridun Vasfi Ulusoy, MD, Department of Cardiology, Ankara Numune Education and Research Hospital, Talatpasa Bulvari, 06100, Sihhiye, Ankara, Turkey.

**Keywords:** Acetylsalicylic acid resistance, Coronary artery disease, Diabetes mellitus

## Abstract

***Objective*** ***:*** Several studies have demonstrated the beneficial role of antiplatelet therapy with acetylsalicylic acid (ASA) at atherosclerotic vascular disease. Antiaggregant effect of ASA is not uniform in all patients. Purpose of the present study is to evaluate the prevalence of ASA resistance in patients with type 2 diabetes mellitus (T2DM), pre-diabetes and non-diabetic coronary artery disease (CAD).

***Methods:*** Effect of ASA was assessed using the platelet function analyzer (PFA-100) system. Resistance to ASA was defined as a normal collagen/epinephrine induced closure time after one week of ASA therapy. Patients with non-diabetic CAD, pre-diabetes and T2DM were compared.

***Results:*** ASA resistance was found in 26 (37.1%), 6 (17.6%) and 41 (26.5%) patients in the groups, respectively (p=0.154). ASA resistance was found to be significantly higher in men, smokers and insulin users, besides this it was found to be significantly lower in beta blocker (BB) users, angiotensin converting enzyme inhibitor (ACEI) users with univariate analysis. However insulin usage was found to be the single effective parameter on ASA resistance in multivariate analysis.

***Conclusion:*** There was no difference with regard to ASA resistance between groups. While ASA resistance was higher in men, smokers and insulin users, it was lower in patients using BBs and ACEIs.

## INTRODUCTION

Acetylsalicylic acid reduces the risk of cardiovascular events in a wide range of patients with established cardiovascular disease (CVD).^1^ Type 2 diabetes is associated with a 2-3 fold increased risk of CVD which is the leading cause of mortality and morbidity.^2^ Clinical guidelines recommend use of ASA for secondary prevention in type 2 diabetic patients with a history of CVD.^3^

Despite therapeutic benefits of ASA, its antiplatelet effect is not uniform in all patients and platelet aggregation studies have shown that 5,5–60% of patients appear to be unresponsive to the antiplatelet effect of ASA.^4^ These patients are clinically referred as ASA-resistant or ASA non-responders. Acetylsalicylic acid resistance has been associated with an increased risk of thrombotic and embolic cardiovascular events compared to patients who are ASA-sensitive.^5^^,^^6^

Hereby, we aimed to compare the frequency of ASA resistance between patients with diabetes mellitus type 2 (T2DM), pre-diabetic patients and non-diabetic coronary artery disease (CAD) patients, and to bring into light the factors that may be related to ASA resistance.

## METHODS

***Study design: ***The study protocol was approved by local ethics committee of Ankara Numune Education and Research Hospital and was conducted in accordance with the Decleration of Helsinki. The effect of ASA was assessed using the platelet function analyzer (PFA-100) system. Patients with abnormal blood count results, those with hepatic and renal disease, and those taking drugs that affect the platelet function were excluded from the study. Resistance to ASA was defined as a normal collagen/epinephrine induced closure time (CTCEPI) (82–165 s) after one week of ASA therapy. Three age- and sex- matched groups including the non-diabetic CAD (group 1, n=70), pre-diabetes (group 2, n=34) and T2DM (group 3, n=155) were compared. We determined pre diabetes as impaired fasting glucose and impaired glucose intolerance.

***Laboratory Assays:***

***PFA-100: ***The PFA-100 is an automated test system which simulates platelet-based hemostasis in vitro. The test cartridge simulates an injured blood vessel and measures the time required to form a platelet plug [defined as closure time (CT)] which occludes a microscopic aperture cut into a collagen/epinephrine- or collagen/ADP-coated membrane under high shear flow condition.^7^

The collagen/epinephrine cartridge is the primary cartridge for detection of the aspirin effect on platelet aggregation. All the blood samples were tested according to the manufacturer’s instruction not earlier than 30 minutes and within two hours of blood sampling. Previous studies^6^ have shown that there is no statistically significant effect of blood sample storage on CT measurements for up to 4 hours. The maximal CT for collagen/epinephrine cartridges is 300 s and values greater than 300 s were reported as non-closure. In these cases, a value of 301 s was assigned for statistical calculations.

***Statistical Analysis: ***Analysis of the data was performed using the Statistical Package for Social Sciences (SPSS) version 13.0 software (SPSS Inc., Chicago, IL, United States). The metric discrete variables were shown as mean ± standard deviation, and percentages were used for the categorical variables. The chi-square tests were used to assess the statistical significance of the differences between the groups in the frequency distribution of categorical variables, unless the expected cell size was less than five, in which case the Fisher’s exact test was used. The medians were compared using the Mann Whitney U test when the number of independent groups was two. The differences between the medians of more than two groups were evaluated using the Kruskal Wallis test. Univariate ve multivariate regression analysis were used for estimating the relationship among variables. A p value of less than 0.05 was considered statistically significant.

## RESULTS

**Fig.1:**
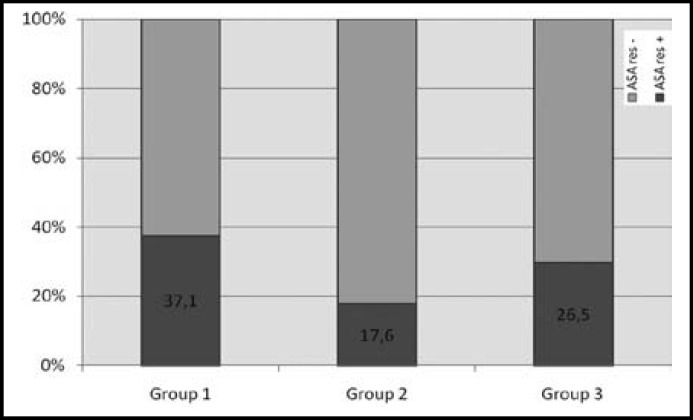
ASA resistance of groups.

The baseline characteristics of groups are shown in Table-I. ASA resistance was found to be similar between groups (p=0.154) (Fig.1). CTCEPI values were also non significant between the groups (219.7±79.9 s, 252.1±60.9 s, 234.7±71.6 s respectively, p=0,121). When the cases with ASA resistance were compared with cases without ASA resistance, no significant differences were encountered with regard to age, duration of T2DM, glycemic and lipid parameters, hCRP, Htc, platelet number, uric acid, fibrinogen and microalbumin. The rate of males in the ASA resistance cases was found to be higher than that in the ASA sensitive cases (58.7 vs 45.6%, p=0.047).

The smoking status and the frequency of hyperlipidemia (HPL) in the ASA resistance cases were higher than those of the non-resistant cases (35.1% vs 26.9%, p=0.025; 68.4% vs 55.0, p=0.037). While ASA resistance was more frequent in those taking insulin (50.0% vs 23.6%, p=0.012) than in those not taking insulin, patients using beta blockers and angiotensin converting enzyme inhibitors (ACEI) had significantly lower ASA resistance (48.3% vs 57.3%, p=0.035; 27.6% vs 37.4%, p=0.029) (Table-II). The mean CTCEPI value in patients taking insulin was 204.4±70.4, and this value in patients not taking insulin was 250.0±63.9 (p=0.002). The effect of insulin use on ASA resistance was seen to continue in multivariate regression analysis. (Table-III).

## DISCUSSION

In the present study we found that there is no difference between groups of diabetic patients, pre-diabetic patients and non-diabetic coronary artery disease patients with regard to ASA resistance. There are some studies comparing ASA resistance between diabetic and coronary heart disease patients, but to our knowledge there is no study evaluating ASA resistance in pre-diabetic patients. The findings of studies assessing ASA resistance in patients with diabetes mellitus or cardiovascular disease prove confusing results. Abaci et al. found that diabetic patients had less ASA resistance than patients with coronary artery disease.^8^ Primary Prevention Project study showed that non-diabetic patients have a 41% decrease in heart disease-related death with 100 mg/day ASA versus 10% in diabetic patients.^9^ The writers concluded that low decrease of end-points at diabetic group might in part be due to ASA resistance. These two studies are contrary examples for ASA resistance in diabetic patients. In our study we could not found any difference between diabetic, prediabetic and non-diabetic coronary artery patients with respect to ASA resistance. Another study had similar findings like ours that there was no relationship between ASA resistance and presence of diabetes in stroke patients.^10^

In this study, while there was ASA resistance in 43.3% of patients taking insulin, there was ASA resistance in 19.1% of those not taking insulin. It is known that diabetic patients have high platelet reactivity.^11^ Although insulin suppresses P2Y_12 _mediated platelet aggregation in healthy subjects,^12^ it is known that if there is insulin resistance such as DM accompanying central obesity, obesity and HT, there is unresponsiveness to the antiaggregant effect of insulin.^12^^-^^14^ In our study, patients who use insulin had significantly higher levels of HbA1c and hCRP levels, higher microalbuminuria, longer mean duration of DM and higher values of fasting glucose, post-prandial glucose. These findings give us that patients using insulin have a higher degree of disease status and inflammatory status. These higher levels of inflammation and hyperglycemia indicators make one think that the reduced sensitivity to aspirin in T2DM may be related to hyperglycaemia or to the associated inflammation.^15^ In a study, a correlation between in vitro TxB2 production and systemic levels of fasting glucose or HbA1c was demonstrated.^16^ The reduced inhibitory action of aspirin on platelet COX-1 may be attributed either to the glycation-induced conformational changes of the platelet membranes with resulting impaired aspirin entrance, and/or to a less-efficient acetylation previously demonstrated in platelets re-suspended in high glucose medium.^17^^,^^18^

In our study, ASA resistance was detected lower in patients taking beta blocker and ACE-I. In a study, patients with effective platelet inhibition were found to be more frequent users of beta-adrenoceptor antagonists and ACE inhibitors, whereas patients with ineffective platelet inhibition were found to be more frequent users of statins.^19^ The effects of beta-adrenoceptor antagonists on collagen and epinephrine-induced platelet aggregation suggest an additive effect of these drugs with respect to antiplatelet therapy. A study has reported that the benefits of ASA and ACE inhibitors may be attenuated when both agents are used together,^20^ but another study has showed no significant effect of ACEI on platelet aggregation on the logistic regression analysis.^21^

We found that smokers have significantly higher ASA resistance. We know that smoking generates a prothrombotic state both with acute usage and long term smoking.^22^^-^^23^ Recurrent stimulation of platelets by smoking may explain the ASA resistance in smokers. Eskandarian et al. demonstrated smoking as a risk factor for ASA resistance at chronic stable angina patients.^24^

There is confusing findings about ASA resistance and gender. While some studies demonstrate higher ASA resistance with male gender, some demonstrate female gender.^8^^,^^25^ We found ASA resistance higher at men in our study. There are arguments that hyperlipidaemia can cause ASA resistance. Friend et al. found ASA resistance was significantly higher in patients with hyperlipidaemia, they also showed the relationship with total cholesterol levels, LDL levels with ASA resistance.^26^ In our study we find hyperlipidemia was significantly higher in ASA resistance cases; but we could not found any relationship with lipid subfractions.

Group 1: Patients with Coronary Artery Disease;

Group 2: Prediabetic Patients; Group 3: Diabetic Patients.

**Table-I:** Clinical and laboratory parameters of the groups

*Variables*	*Group 1* *(n=70)*	*Group 2* *(n=34)*	*Group 3* *(n=155)*	*p*
Age, years	57.0±7.6	55.0±10.7	54.5±9.0	0.157
Gender (M/F)	28/42	14/20	63/92	0.599
BMI	29.4±6.4	31.0±4.7	30.6±5.4	0.312
Fasting glucose, mg/dl	85.5±8.4	101.0±16.2	163.5±70.0	*<0.001*
Postprandial glucose, mg/dl	98.7±15.2	129.2±29.1	230.1±95.7	*<0.001*
HbA1c, %	5.6±0.4	6.2±0.8	8.9±2.4	*<0.001*
TC, mg/dl	172.2±42.4	179.4±51.4	182.4±54.0	0.404
TG, mg/dl	146.4±93.5	125.4±57.0	166.1±159.3	0.288
HDL-C, mg/dl	36.5±9.6	41.9±9.5	40.4±10.5	*0.015*
LDL-C, mg/dl	105.9±34.1	124.0±44.5	112.5±41.7	0.131
Htc, %	43.5±4.0	40.0±4.2	41.2±5.1	*0.001*
Plt, x1000/mm^3^	276.3±31.2	267.3±63.9	258.3±94.0	0.849
Uric acide, mg/dl	5.8±1.7	4.7±1.4	4.8±1.3	*<0.001*
hCRP, mg/dl	2.3±2.4	2.0±1.8	3.6±3.5	*0.013*
Hcy, µmol/L	18.1±9.7	12.6±4.6	12.4±6.2	*<0.001*
Fibrinogen, mg/dl	349.2±67.8	338.5±60.8	370.5±84.0	0.696
Microalbumin, mg/24 hour	3.5±5.5	9.5±10.2	67.7±274.5	0.158
CTCEPI, s	219.7±79.9	252.1±60.9	234.7±71.6	0.121

BMI: body mass index; CTCEPI: collagen/epinephrine induced closure time;

HbA1c: glycosylated hemoglobin; hCRP: high-sensitivity C-reactive protein; Hcy: hornocysteine;

HDL-C: high density lipoprotein – cholesterol; Htc: haematocrit;

LDL-C: low density lipoprotein-cholesterol; Plt: platelets; TC: total cholesterol; TG: triglyceride;

Group 1: Patients with Coronary Artery Disease; Group 2: Prediabetic Patients; Group 3: Diabetic Patients.

**Table-II:** Comparison of the demographic and laboratory parameters between those with ASA resistance and those without

*Variables*	*ASA resistance + (n=73)*	*ASA resistance - (n=186)*	*P*
Age, years	56.0±7.4	54.8±9.5	*0.492*
DM duration, years	9.4±6.5	7.4±6.5	*0.976*
Fasting glucose, mg/dl	125.6±66.4	132.8±64.5	*0.144*
Postprandial glucose, mg/dl	197.2±73.6	196.4±91.4	*0.748*
HbA1c, %	8.7±1.8	8.7±2.7	*0.976*
TC, mg/dl	181.2±47.5	178.6±49.6	*0.888*
TG, mg/dl	159.3±121.1	158.5±150.0	*0.950*
HDL-C, mg/dl	39.3±10.5	39.0±9.4	*0.907*
LDL-C, mg/dl	112.3±39.9	111.9±39.3	*0.701*
Hb, g/dl	14.2±1.6	14.1±1.6	*0.568*
Plt, x1000/mm^3^	247.2±94.0	252.9±83.7	*0.088*
Htc, %	41.5±4.5	42.0±5.2	*0.239*
MPV, fl	9.0±0.9	8.7±0.9	*0.226*
Uric acid, mg/dl	5.4±1.6	5.0±1.6	*0.273*
hCRP, mg/dl	3.3±3.6	2.6±2.7	*0.504*
Hcy, µmol/L	16.0±10.2	14.3±7.1	*0.195*
Fibrinogen, mg/dl	359.1±74.2	356.3±78.1	*0.864*
Microalbumin, mg/24 hour	8.6	9.6	*0.855*
Male Gender (%)	58,7	45,6	*0.047*
Smoking, %	35,1	26,9	*0.025*
HT, %	64,9	59,7	*0.500*
HPL, %	68.4	55.0	*0.037*
Insulin, %	50.0	23.6	*0.012*
MET, %	46.2	51.4	*0.647*
SU, %	26.9	30.6	*0.728*
TZD, %	7.7	5.6	*0.697*
Beta blocker, %	48.3	57.3	*0.035*
ACEI, %	27.6	37.4	*0.029*
ARB, %	34.5	26.9	*0.293*
CCB, %	12.1	16.2	*0.467*
Statin, %	47.4	43.8	*0.656*

DM: diabetes mellitus; Hb: hemoglobin; HbA1c: glycosylated hemoglobin;

hCRP: high-sensitivity C-reactive protein; Hcy: hornocysteine;

HDL-C: high density lipoprotein – cholesterol; Htc: haematocrit;

LDL-C: low density lipoprotein-cholesterol; MPV: mean platelet volume; Plt: platelets;

TC: total cholesterol; TG: triglyceride; ACEI: angiotensin-converting enzyme ınhibitor;

ARB: angiotensin receptor blocker; CCB: calcium channel blockers; HT: hypertension;

MET: metformin; SU: sulfonylurea; TZD: thiazolidinediones.

**Table-III:** Multivariate regression analysis of various parameters with collagen/epinephrine induced closure time

*Variables*	*Unstandardized Coefficients*		*Standardized Coefficients*	*t*	*Sig.*
	*B*	*Std. Error*	*Beta*		
(Constant)	0.645	0.389		1.657	0.103
Age	0.006	0.007	0.109	0.882	0.381
Gender	-0.089	0.137	-0.098	-0.648	0.519
Smoking	-0.042	0.151	-0.043	-0.279	0.782
Hypertension	0.112	0.115	0.123	0.972	0.335
Duration of DM	-0.005	0.010	-0.072	-0.525	0.602
Insulin	-0.260	0.126	-0.279	-2.071	*0.043*

## CONCLUSION

We could not find any difference between diabetic, pre-diabetic and non-diabetic coronary artery disease patients with regard to ASA resistance. ASA resistance was determined to be low in patients taking beta blockers and ACE-I than in those not taking these drugs, and higher in those taking insulin, men, and smokers. ASA resistance predictors and related situations should be well defined with larger trials because it is known that ASA resistance increases cardiovascular mortality.

## Notes

***Conflict of Interest, funds, support:*** None declared.
